# Long-read sequencing-based in silico phage typing of vancomycin-resistant *Enterococcus faecium*

**DOI:** 10.1186/s12864-021-08080-5

**Published:** 2021-10-23

**Authors:** Paola Lisotto, Erwin C. Raangs, Natacha Couto, Sigrid Rosema, Mariëtte Lokate, Xuewei Zhou, Alexander W. Friedrich, John W. A. Rossen, Hermie J. M. Harmsen, Erik Bathoorn, Monika A. Chlebowicz-Fliss

**Affiliations:** 1 Department of Medical Microbiology and Infection Prevention, University of Groningen, University Medical Center Groningen, Groningen, the Netherlands; 2 The Milner Centre for Evolution, Department of Biology and Biochemistry, University of Bath, Bath, UK; 3 Department of Pathology, University of Utah School of Medicine, Salt Lake City, UT USA; 4 IDbyDNA Inc., Salt Lake City, UT USA

**Keywords:** MinION, Outbreak, VRE, Typing, Prophage, Molecular epidemiology

## Abstract

**Background:**

Vancomycin-resistant enterococci (VRE) are successful nosocomial pathogens able to cause hospital outbreaks. In the Netherlands, core-genome MLST (cgMLST) based on short-read sequencing is often used for molecular typing. Long-read sequencing is more rapid and provides useful information about the genome’s structural composition but lacks the precision required for SNP-based typing and cgMLST. Here we compared prophages among 50 complete *E. faecium* genomes belonging to different lineages to explore whether a phage signature would be usable for typing and identifying an outbreak caused by VRE. As a proof of principle, we investigated if long-read sequencing data would allow for identifying phage signatures and thereby outbreak-related isolates.

**Results:**

Analysis of complete genome sequences of publicly available isolates showed variation in phage content among different lineages defined by MLST. We identified phage present in multiple STs as well as phages uniquely detected within a single lineage. Next, in silico phage typing was applied to twelve MinION sequenced isolates belonging to two different genetic backgrounds, namely ST117/CT24 and ST80/CT16. Genomic comparisons of the long-read-based assemblies allowed us to correctly identify isolates of the same complex type based on global genome architecture and specific phage signature similarity.

**Conclusions:**

For rapid identification of related VRE isolates, phage content analysis in long-read sequencing data is possible. This allows software development for real-time typing analysis of long-read sequencing data, which will generate results within several hours. Future studies are required to assess the discriminatory power of this method in the investigation of ongoing outbreaks over a longer time period.

**Supplementary Information:**

The online version contains supplementary material available at 10.1186/s12864-021-08080-5.

## Background

The prevalence of vancomycin-resistant *Enterococcus faecium* (VRE*fm*) carriage among hospitalized patients has increased dramatically over the past 15 years. In many parts of Europe, this has resulted in a rapid increase in infections with vancomycin-resistant enterococci (VRE) [1]. This is mainly due to the nosocomial spread of VRE*fm* lineages that are highly adapted to survive in the hospital environment [2]. This is worrisome for several reasons. VRE may cause severe infections in immune-compromised patients, such as patients with hematologic disorders and solid organ transplant patients [3]. Since there is a minimal choice of alternative antibiotic treatment options, VRE has been categorized as a high priority pathogen in the WHO list of antibiotic-resistant pathogens for which the development of new antibiotics is required [4].

To control the spread of VRE*fm*, it is recommended to treat patients carrying VRE*fm* with barrier precautions, trace contacts, and type the isolates [5]. Patients who carry VRE*fm* can develop infections with this pathogen later [6]. Therefore, it makes sense to prevent the transmission of VRE by screening for carriage [7]. Molecular typing of isolates shows whether they are closely related and thereby indicates transmission or not. In case genetically related isolates are identified, contact investigations are initiated. The typing methods used must have a rapid turnaround time for a fast response in outbreak management. Next to this, high discriminatory power is required for VRE*fm* typing. Due to the rapid emergence of nosocomial VRE*fm* lineages, the chromosomal diversity within these lineages is relatively low. This makes it difficult to rule-in isolates that are involved in an outbreak. Even SNP-based analysis of next generating sequencing data may lack power to prove transmission. Nonetheless, core-genome MLST (cgMLST) based on short-read sequencing is recommended for molecular typing of VRE*fm*, since it is a validated method with higher discriminatory power compared to other typing methods as PFGE and MLST [8, 9]. The cgMLST typing results of isolates can be generated in 24–72 h, counting from the moment of DNA extraction [10].

Long-read sequencing is faster and provides useful information about the structural composition of the genome. Results can be generated within minutes [11]. It can be used to detect VRE through metagenomics [11] and for MLST and resistance gene detection in whole-genome sequences of isolates [12]. A drawback of MinION long-read sequencing is the higher error rate when compared to MiSeq short-read sequencing. Since around 8% of bases are miscalled, nanopore sequencing thus far lacks the precision required for SNP-based typing methods [13]. Information about the structural genome composition, however, can be useful for typing as well. Mobile genetic elements may be reliably identified in nanopore sequencing data. This is relevant for VRE outbreaks since recent studies have shown that the type of transposons and their insertion sites in the genome can be specific for an outbreak clone [14–16].

The characterization of bacteriophage content may also be useful for typing. The use of phages in the typing of bacteria had been used in the past. Back in the 1930s, Salmonella species were typed by studying which phages were able to infect the specific strains and to which phages the isolate is resistant [17]. Later on, this phage typing method was also developed for many other pathogens such as *Staphylococcus aureus* [18] and Enterococcus species [19] and used for outbreak investigations. It was noticed that the resistance pattern to infection by bacteriophages was related to the presence of certain prophages [20]. The presence of specific prophage gives immunity to the infections caused by a similar group of bacteriophages. Thus, the conventional phage typing was an indirect detection method for strain-specific prophages. The recent developments in long-read sequencing allow for direct and more robust sequence reconstruction of prophages and their integration sites in bacterial genomes [21]. Thus, direct sequencing information about phage content is more informative than that derived by conventional phage typing. The gain and loss of phages are critical drivers in the differentiation of subtypes with strain-specific properties, even in lineages that are highly similar in their core genome [22]. This leads to our hypothesis that long-read-based characterization of phage content may have high discriminatory power in identifying outbreak strains.

Here, we compared prophages among complete genome sequences of genetically diverse *E. faecium* isolates to explore whether a signature of phage content would be usable for typing of this pathogen. Next, as a proof of principle, we investigated if long-read sequencing data allows for identifying such signatures in clinical outbreak situations by using data from well described outbreak strains [15, 16].

## Results

### Prophage profiles of the 50 publicly available *E. faecium* genomes

In the first part of this study, we investigated the presence of prophages among complete genomes of *E. faecium* isolates from different geographical and genetic backgrounds. In this analysis, we aim to identify prophage patterns that are associated with specific lineages.

**Fig. 1:**
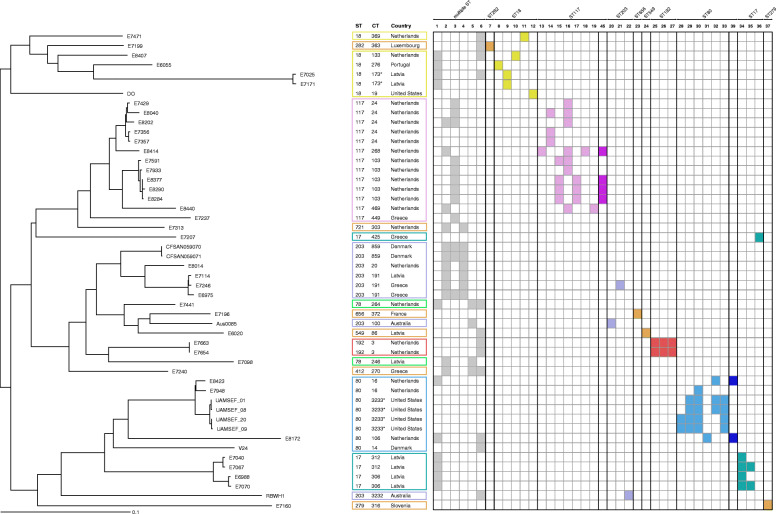
Neighbour joining tree based on cgMLST (1423 target genes) of the publicly available isolates. The sequence types have been highlighted with different colours to differentiate the lineages. The presence and absence of prophages identified in each sequence type is depicted with coloured and white cells, respectively. Isolates marked with an asterisk were manually assigned to the same CT according to the clusters identified in the minimum spanning tree

In Fig. 1, we show the phylogenetic relatedness of 50 *E. faecium* genomes from hospitalised patients, belonging to different sequence types of clonal complex 17 (CC17). For additional information regarding the phylogenetic relationship of these strains, we refer to Additional file 2 where a minimum spanning tree indicates the allelic differences between the isolates.

A variable number of prophages were identified in individual isolates, with a maximum of five prophage regions per genome. When compared to the NCBI viral database, no significant alignments were found that exceeded 10% coverage. An overview of the phages detected in the investigated isolates and their sequence annotations, are presented in Additional files 1 and 3. Six phages (Phage 1 to 6) were detected in multiple lineages while the majority of the phages (Phage 7 to 37) were uniquely present within a single sequence type. The phylogenetic analysis (Additional file 4) revealed that these phages cluster in two major groups, and one phage clustered separately. The first group comprises five phages, which are closest related to the *Siphoviridae* Bacillus SPbeta and Staphylococcus phage SPbeta-like. The second group includes the majority of the phages (*n* = 44) which are closest related to a clade of unclassified *Siphoviridae*, mainly detected in *Listeria* spp. Only Phage 17 is recognized as distinct phage, that is closest relative to the Enterococcus phage IME_EF of the *Siphoviridae* family.

From this analysis, we learned that it is not feasible to type VRE isolates using a single prophage as a reference for typing of outbreak events, given the high diversity in prophage content among different lineages. Instead, it seemed useful to characterise the total prophage content of the index isolate of any given outbreak and compare sequences of other isolates to such unique prophage signature to investigate outbreak events.

### Twelve *E. faecium* isolates for the use of in silico phage typing

In the second part of the study, we applied in silico prophage typing to 12 nosocomial isolates from the UMCG. These isolates belonged to two different sequence and complex types, namely ST117, CT24 and ST80, CT16. Epidemiological data related to these isolates is summarised in Table 1.

**Table 1:** Information on the UMCG isolates used in this study

ID	V1	V2	BL4	BL5	BL6	B2	B11	A7	BL1	BL2	BL3	S1
ST	80	80	80	80	80	117	117	117	117	117	117	117
CT	16	16	16	16	16	24	24	24	24	24	24	24
Vancomycin Resistance	VRE	VRE	VSE	VSE	VSE	VRE	VRE	VRE	VSE	VSE	VSE	VSE
Year of collection	2019	2019	2016	2015	2014	2017	2017	2014	2017	2015	2014	2016
Phage 38	1	1	0	0	0	0	0	0	0	0	0	0
Phage 39	1	1	1	1	0	0	0	0	0	0	0	0
Phage 40	1	1	1	1	1	0	0	0	0	0	0	0
Phage 41	0	0	0	0	1	0	0	0	0	0	0	0
Phage 42	0	0	0	0	0	1	1	1	1	1	1	1
Phage 43	0	0	0	0	0	1	1	1	1	1	1	1
Phage 44	0	0	0	0	0	1	0	0	1	1	1	1
Phage 45	0	0	0	0	0	0	0	1	0	0	0	0
Phage 40.1	1	1	1	1	1	1	1	1	1	1	1	1
Phage 42.1	0	0	0	0	0	1	1	1	1	1	1	1
Phage 44.1	0	0	0	0	0	1	1	0	0	0	0	0

*Abbreviations*: *ID* identification, *ST* sequence type, *CT* complex type.

**Fig. 2:**
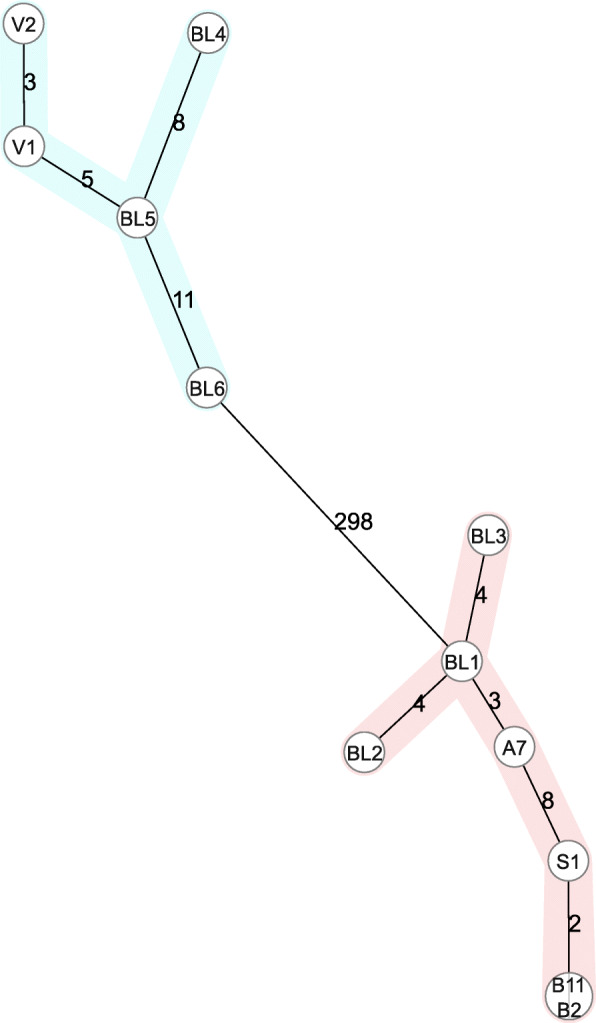
Minimum spanning tree based on cgMLST (1423 target genes) of MiSeq data of 12 *E. faecium* isolates. The numbers next to the lines correspond to the allele differences between isolates. CT16 and CT24 are depicted in blue and red, respectively

To assess these isolates’ phylogenetic relatedness, cgMLST was performed on the MiSeq data (Fig. 2). Among isolates of CT16 we detected 11 allele differences, when considering samples collected in the same outbreak, only 3 allele differences were identified. In isolates from CT24, we detected a maximum of 8 allele differences.

**Fig. 3:**
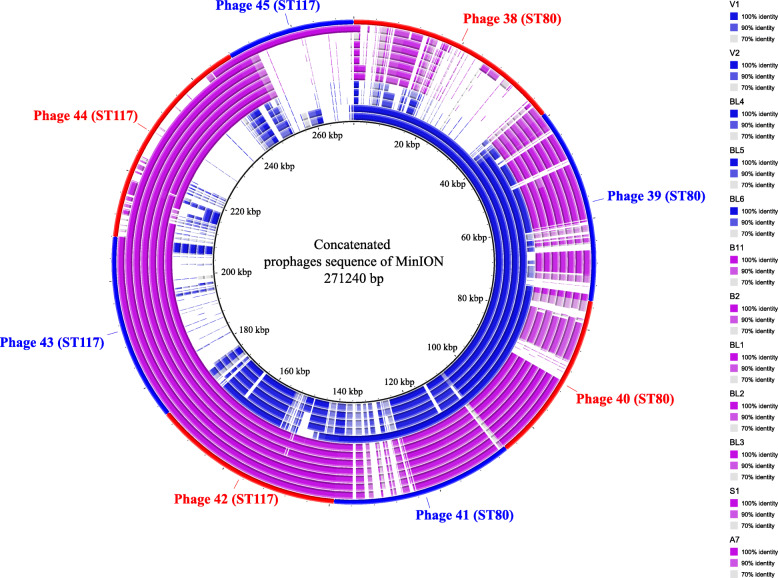
Concatenated prophage sequences identified in UMCG isolates. The concatenated reference sequence is indicated as the red-blue outer circle, the inner circles represent the UMCG isolates. CT16 and CT24 isolates are depicted in dark-blue and purple, respectively. The isolates in the legend should be read starting from top to down and from inner circle to outer circle

Among investigated UMCG isolates, a total of eight prophages (phage 38–45) were identified based on long-read data. These were used to create a concatenated reference sequence for the BLAST analysis presented in Fig. 3 and summarised in Table 1. To verify that the profile observed was unique for UMCG isolates, this reference phage sequence was compared to the publicly available isolates. Phage 38 and 41 were uniquely present in UMCG isolates V1, V2 and BL6, respectively. Phage 39 and 45 were also present in genomes of publicly available isolates belonging to the same sequence type (Fig. 1). Phages 40, 42, 43 and 44 presented a high similarity with phages 17, 22, 3 and 24, respectively.

**Fig. 4:**
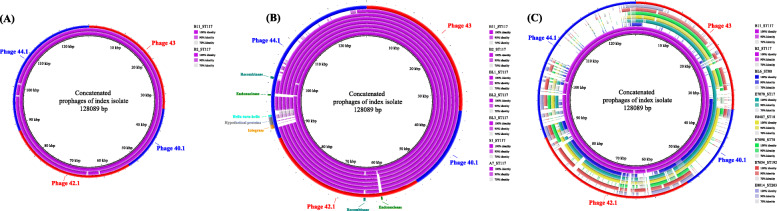
(A-C): Clinical application of prophage typing during an outbreak investigation. The concatenated prophage reference sequence is indicated as red-blue outer circle. In panel A, the first inner circle from the centre represents the sequence of an outbreak isolate (index patient) belonging to ST117/CT24. The second circle from the centre represents the sequence of an additional isolate which belongs to the same outbreak event and complex type. In panel B, next to the isolates indicated in panel A, isolates belonging to the same complex type, but not epidemiologically related to the same outbreak event are presented. In the last outer ring, the genes responsible for the differences between the isolates has been annotated. In panel C, sequences of additional isolates belonging to different sequence types than the outbreak event are depicted in different colours. The isolates in the legend should be read starting from top to down and from inner circle to outer circle

Next, as a proof-of-principle, we tested the use of long-read sequencing based in silico phage identification for the typing of *E. faecium* in an outbreak investigation. We exclusively used isolate B11 which belonged to the index patient of an outbreak event and identified the phages that characterise this isolate (Phage 43, 40.1, 42.1, 44.1). We then used these phages to generate a concatenated reference sequence and compared it to isolate B2, collected during the same outbreak episode (Fig. 4A). Despite some minor gaps, the sequence coverage of the reference was 98%, indicating a high similarity between isolates. We investigated in which functional module of the predicted prophage sequence the main differences occurred among the closely related isolates of ST117/CT24 presented in Fig. 4B. This revealed that all the isolate which did not belong to the 2017 outbreak differed in the *integrase* gene, and in an adjacent cluster of hypothetical protein genes. Isolate B2 slightly differed from the reference in the endonuclease and recombinase genes. In this set of isolates, the median reference coverage was found to be lowered to 96%. Interestingly, one isolates (A7) collected during a different outbreak showed a low similarity (78%) due to the lack of phage 44.1. This classification is in line with the cgMLST analysis presented in Fig. 3 and with the SNP-based analysis presented in Additional file 5. To further evaluate our method, we expanded the analysis by including 57 isolates collected from VREfm outbreaks that occurred in 2014 and 2017. The data presented in Additional file 6 confirmed that isolates collected during 2017 outbreak belonging to ST117/CT24 lineage showed the highest similarity to the reference isolate B11 (97–100% coverage). Instead, the isolates collected in 2014 outbreak and classified as ST117/CT24 did not show a concordant profile, having a median reference coverage of 79%. Comparable or even lower similarity was observed when isolates obtained from 2014 outbreak but belonging to different lineages, e.i. ST80/CT104 (50%) and ST117/CT103 (79%), were included. When representative isolates belonging to different sequence types and collected outside our hospital were used for the comparison (Fig. 4C), the median coverage was all < 70%, underscoring their unrelatedness. The analysis of the complete list of isolates is presented in Additional files 7 and 8.

Finally, we evaluated the proposed phage typing method using short-reads data in isolates typed as ST80/CT104, collected during an outbreak event in 2014. In this lineage, two phages were identified and compared to the same 57 investigated isolates where the median coverage for this reference was 95%. Using this approach all outbreak isolates belonging to ST80/CT104 were correctly identified with a coverage of 100% (Additional file 9). Nearly all isolates of other tested outbreaks showed coverage below 97%. The main differences observed between the reference and other isolates were linked to some elements of the phage structure related to the replication, tail, packing and lysogeny modules (Additional file 10). However, a cluster of isolates belonging to a different complex type (ST117/CT103) showed phage sequences with high percentages coverage (96–100%), unexpectedly.

According to our results, a threshold of 97% coverage could be considered, allowing to rule-out clonal transmission. The phage typing method cannot be used for a definitive inclusion of isolates into an outbreak cluster, since we found a cluster of false positive matches from a different lineage. The phage typing method provides complementary results to typing by cgMLST, and can therefore be used as tool to discriminate between isolates that belong to the same complex type, but are not epidemiologically related.

## Discussion

*Enterococcus faecium* has been widely recognised as a potential threat for hospitalised patients, especially immune-compromised, due to the multidrug resistance profile characterizing this opportunistic pathogen [4]. Early detection of VRE is a crucial element in preventing hospital outbreaks [2]. In this scenario, the use of next-generation sequencing (NGS) has been proven successful and allowed to track the transmission route with variable turnaround time depending on the technology used [10]. In this study, we applied in silico phage typing using MinION long-read sequencing as an alternative approach for the early detection and screening of VRE*fm* outbreaks.

In our analysis, a variable number of prophages were found to be integrated in the genome of different *E. faecium* lineages. This is in line with previous observations indicating that bacterial genomes contain up to 20% of prophage genes [23]. Van Schaik et al. estimated that for *E. faecium* strains, between 2 and 5% of the total number of CDS could belong to predicted phages [24]. To the best of our knowledge, no other similar research was performed, indicating that this is the first study to identify prophage signatures as a typing method for *E. faecium*. Our analysis of publicly available isolates revealed the presence of prophages shared between isolates belonging to different lineages and unique profiles present within the same lineage.

The application of solely long-read sequencing for the assembly of complete genomes has proved successful for different microorganisms [21, 25], and recently the MLST genotyping of *E. faecium* using ONT technology was suggested in hospital infection control [12]. Our study confirmed the possibility of *E. faecium* full genome reconstruction using only long-read data which supports the possibility to identify a prophage signature, possibly consisting of multiple phages for a higher discriminatory power, that could be applied during an outbreak event as a typing method to identify if the isolates are related. Our analysis on long-read sequencing data indicates that, when comparing isolates sharing the same genetic background as predicted by cgMLST, a high degree of similarity can be found. Contrary, if different lineages are compared, a clear distinction of prophage profile can be observed. This indicates that, in a situation where clonal spread is present, a shared prophage reference could identify related isolates and distinguish them from unrelated ones. We observed that using only two phages as reference can be a limitation of this method, not allowing to rule out clonal transmission especially when comparing isolates collected during a long period of time. Recent horizontal transfer of bacteriophages also may result in the detection of identical phage sequences in different lineages. None of the cgMLST, SNP-based and the in silico phage typing methods have the discriminatory power to prove clonal transmission conclusively, however, the results of all these methods provide complementary information, and would need to be interpreted together with epidemiological data.

Interestingly, we detected genetic diversity in the integrase gene in closely related isolates belonging to CT24. In literature [26, 27], this gene was described as a potential target for typing and as an indicator of genomic diversity, making it a useful target to distinguish between closely related strains within hyperepidemic lineages.

The application of third-generation sequencing, such as ONT technology, to clinical microbiology and infection prevention is of great potential [28]. The fast turnaround time and the possibility to analyse preliminary data in real-time make this technology a promising approach for outbreak investigations where a quick reaction is essential [25]. Computed prediction of prophage regions, and direct detection of these regions on the sequence reads would strongly shorten the turnaround time. In that case, we estimate that results could be generated in less than 2 h. We underline that applying this technique would be more suitable in short-term outbreak investigation considering the variability in phage content that can occur over time. An example of such scenario was isolate A7 which, due to the lack of phage 44.1, had a lower similarity to the reference compared to other isolates of the same CT. Moreover, we acknowledge that the use of only long-read sequencing data should be further developed and tested in future studies with a larger sample size to validate our results.

## Conclusions

In conclusion, we show a proof of principle for in silico prophage typing. Based on this method, we were able to group CT24 *E. faecium* isolates and distinguish them from different complex types. Using this approach we correctly identified isolates belonging to the same outbreak event, which is not always possible based on cgMLST. Long-read sequence-based phage typing is promising as a rapid typing method with high discriminatory power, and it seems worthwhile to assess this in future validation studies.

## Methods

### *E. faecium* isolates

For this study, 50 hybrid assembled *E. faecium* genomes were downloaded from NCBI or the publicly available Gitlab repository (https://gitlab.com/sirarredondo/efaecium_population/tree/master/Files/Unicycler_assemblies). These isolates were collected from hospitalised patients, and their metadata is summarised in Additional file 1.

Additionally, twelve *E. faecium* isolates were used, collected at the University Medical Center Groningen (UMCG) between 2014 and 2019. Five of these isolates (A7, B2, B11, V1, V2) were collected during three different outbreak events in 2014, 2017, and 2019. The other isolates were either recovered from independent bloodstream infections (BL1-BL6) or surveillance procedures (S1). These *E. faecium* isolates were initially sequenced with Illumina MiSeq and retrospectively assessed by Oxford Nanopore Technologies (ONT) sequencing (MinION).

Finally, VREfm isolates collected in two outbreak events which occurred in 2014 and 2017 and previously described by Zhou et al. and Lisotto et al. have been included in this study to further validate the method proposed [15, 16].

### DNA preparation and sequencing

Twelve bacterial isolates were grown overnight on blood agar plates at 37 °C. Bacterial DNA isolation was performed using the DNeasy UltraClean Microbial Kit (MO BIO Laboratories, Carlsbad, CA) following the manufacturer’s protocol. DNA concentration and purity were measured by the Qubit dsDNA HS and BR assay kit (Life Technologies, Carlsbad, CA, USA). Initially, these isolates were sequenced by short-read sequencing with Illumina MiSeq (Illumina, San Diego, CA, USA). DNA libraries were prepared with the Nextera XT v2 kit (Illumina). These isolates were also sequenced with the MinION (ONT, Oxford, UK). Libraries were prepared using the Rapid Barcoding Sequencing kit, SQK-RBK004 (ONT). The library was loaded on a FLO-MIN106D flow cell R9.4.1 (ONT) and sequenced on a MinION device for 48 h. Base-calling was performed using Guppy v2.1.3 (ONT).

### Data analysis

De novo assemblies of short-read sequencing were performed in CLC Genomics Workbench v12 (QIAGEN, Hilden, Germany). The adapters of the long-reads were trimmed and demultiplexed by using Porechop v0.2.4 (https://github.com/rrwick/Porechop). Long-read assemblies were performed by Flye v2.5 [29]. The cgMLST typing was performed using Ridom SeqSphere+ software v6.0.3 (Ridom GmbH, Münster, Germany). Snippy v4.3.0 (https://github.com/tseemann/snippy) was used to identify core genome concatenated single nucleotide polymorphisms (SNPs) in the short-read sequenced isolates using *E. faecium* E1 strain (NZ_CP018065.1) as reference.

### Phage identification

The presence of prophages was investigated using the online tool PHASTER [30]. The predicted prophage regions were manually extracted from the genomic sequences and were investigated in detail using Artemis [31]. We investigated the genome of *E. faecium,* considering the direction of the open reading frames genes in the predicted prophage regions as suggested before [23, 32]. In the outbreak investigation, a concatenated reference sequence was created containing the prophage genomes identified in *E. faecium* isolate of the index patient. Subsequently, a BLAST analysis was performed to compare the reference against the long-read sequencing data of other isolates. In this analysis we accepted some degree of sequence discrepancy when comparing assemblies generated from long-read sequencing data. The results were visualised using BRIG [33]. Moreover, ViPTree [34] was used to classify the bacteriophages in a genome-wide approach.

## Supplementary Information

**Additional file 1.** Accession number and information on publicly available isolates.

**Additional file 2.** Minimum spanning tree based on cgMLST (1423 target genes) of the publicly available isolates. Numbers within the circle indicate the isolates. The numbers next to the lines correspond to allele differences between the isolates. The different colours highlight groups of closely related isolates.

**Additional file 3.** Sequence annotation of the prophages regions identified in the isolates investigated in the study.

**Additional file 4.** Dendrograms revealing genomic similarity in the publicly available isolates.

**Additional file 5 **SNP-based analysis of the short-read data from UMCG isolates. *E. faecium* E1 strain (NZ_CP018065.1) was used as reference.

**Additional file 6.** Analysis of the UMCG outbreak isolates against concatenated reference sequence of index isolate (B11). The concatenated prophage reference sequence is indicated as red-blue outer circle. The isolates in the legend should be read from top to down and from inner circle to outer circle.

**Additional file 7.** Analysis of the publicly available isolates against the concatenated reference sequence of index isolate (B11). The isolates in the legend should be read from top to down and from inner circle to outer circle.

**Additional file 8.** Percentage of similarity between the publicly available genomes and the concatenated reference sequence of index isolate (B11).

**Additional file 9.** Percentage of similarity between the outbreak isolates and the reference sequence of ST80/CT104 lineage.

**Additional file 10.** Analysis of the outbreak isolates against the reference sequence of ST80/CT106 lineage. The isolates in the legend should be read from top to down and from inner circle to outer circle.

## Data Availability

Illumina MiSeq and Oxford Nanopore Technologies MinION reads of the 12 *E. faecium* isolates collected at UMCG and used in this study have been deposited in the European Nucleotide Archive (ENA) public project PRJEB41626 (https://www.ebi.ac.uk/ena/browser/view/PRJEB41626). Isolate A7 can be retrieved under the BioProject no PRJEB25590; BioSample SAMEA104703752 (https://www.ebi.ac.uk/ena/browser/view/SAMEA104703752).

## Abbreviations

VRE: Vancomycin-resistant enterococci
VRE*fm*: Vancomycin-resistant *Enterococcus faecium*
cgMLST: Core-genome Multi Locus Sequence Type
UMCG: University Medical Center Groningen
ONT: Oxford Nanopore Technologies
E1: *E. faecium* strain (NZ_CP018065.1)
CC17: Clonal complex 17
CT: Complex type
ST: Sequence type
NGS: Next-generation sequencing
